# C11ORF24 Is a Novel Type I Membrane Protein That Cycles between the Golgi Apparatus and the Plasma Membrane in Rab6-Positive Vesicles

**DOI:** 10.1371/journal.pone.0082223

**Published:** 2013-12-02

**Authors:** Vincent Fraisier, Amal Kasri, Stéphanie Miserey-Lenkei, Jean-Baptiste Sibarita, Deepak Nair, Adeline Mayeux, Sabine Bardin, Yusuke Toyoda, Ina Poser, Andrei Poznyakovskiy, Bruno Goud, Anthony A. Hyman, Ariane Dimitrov

**Affiliations:** 1 UMR144, Institut Curie/ CNRS, Cell and Tissue Imaging Platform, Paris, France; 2 UMR144, Institut Curie/CNRS, Molecular Mechanisms of Intracellular Transport, Paris, France; 3 University of Bordeaux, Interdisciplinary Institute for Neuroscience, Bordeaux, France; 4 CNRS, UMR 5297, Bordeaux, France; 5 Max Planck Institute of Molecular Cell Biology and Genetics, Dresden, Germany; University of Nebraska Medical Center, United States of America

## Abstract

The Golgi apparatus is an intracellular compartment necessary for post-translational modification, sorting and transport of proteins. It plays a key role in mitotic entry through the Golgi mitotic checkpoint. In order to identify new proteins involved in the Golgi mitotic checkpoint, we combine the results of a knockdown screen for mitotic phenotypes and a localization screen. Using this approach, we identify a new Golgi protein C11ORF24 (NP_071733.1). We show that C11ORF24 has a signal peptide at the N-terminus and a transmembrane domain in the C-terminal region. C11ORF24 is localized on the Golgi apparatus and on the *trans-*Golgi network. A large part of the protein is present in the lumen of the Golgi apparatus whereas only a short tail extends into the cytosol. This cytosolic tail is well conserved in evolution. By FRAP experiments we show that the dynamics of C11ORF24 in the Golgi membrane are coherent with the presence of a transmembrane domain in the protein. C11ORF24 is not only present on the Golgi apparatus but also cycles to the plasma membrane *via* endosomes in a pH sensitive manner. Moreover, via video-microscopy studies we show that C11ORF24 is found on transport intermediates and is colocalized with the small GTPase RAB6, a GTPase involved in anterograde transport from the Golgi to the plasma membrane. Knocking down C11ORF24 does not lead to a mitotic phenotype or an intracellular transport defect in our hands. All together, these data suggest that C11ORF24 is present on the Golgi apparatus, transported to the plasma membrane and cycles back through the endosomes by way of RAB6 positive carriers.

## Introduction

The Golgi complex plays a central role in multiple functions essential for cell growth, homeostasis and division. It processes and sorts proteins and lipids synthesized in the endoplasmic reticulum and connects the anterograde and retrograde trafficking pathways. Golgi function is associated with its unique ultrastructural characteristics.

The Golgi apparatus is composed of stacks of flat cisternae. In mammalian cells, a large number of stacks are laterally connected to form a ribbon-like structure close to the microtubule organizing center [1,2]. Each stack exhibits an internal polarity: the *cis* part exchanges material with the endoplasmic reticulum through the intermediate compartment whereas, at the other extremity the trans-Golgi separated from the *cis*-Golgi by medial cisternae is in contact with the trans-Golgi network (TGN). The TGN is responsible for the final sorting steps that target several different destinations and exchanges material with the endocytic compartments.

As is the case for all organelles, the Golgi apparatus is divided between the two daughter cells during mitosis. However for the Golgi apparatus, division occurs by complete dispersion in the mother cell before mitosis [3]. Dispersion starts in G2 when the Golgi apparatus fragments by disruption of the ribbon [4,5]. Recent work has linked Golgi apparatus fragmentation to cell cycle progression. Indeed, cells do not enter mitosis if the ribbon is not disrupted [6]. This control was described as the “Golgi mitotic checkpoint”. However, signaling pathways and/or mechanisms by which Golgi apparatus fragmentation and cell cycle progression are linked are still unclear. Further studies on Golgi proteins have shown a potential relationship between the Golgi apparatus and the mitotic apparatus [6]. Two proteins involved in the stacking of the Golgi cisternae, GRASP65 (Golgi Reassembly protein of 65KDa) and GM130 (Golgi Matrix protein of 130KDa), seem to play a role in the proper formation of the mitotic spindle [7]. On the other hand several Golgi proteins were shown to play a direct role in cell cycle regulation. For example after knockdown of the small GTPase RAB6A’ by RNAi the Mad2 checkpoint is activated leading to a metaphase block [8]. The detailed roles of these proteins in cell cycle progression have only started to be investigated. Thus we only have a vague idea of how the function and organization of the Golgi apparatus is linked to cell cycle progression.

In order to improve our knowledge on the role of the Golgi apparatus in cell cycle regulation, we have combined the results of two recent studies. The first one reported a genome-scale RNA-mediated interference screen in HeLa cells designed to identify human genes that are important for cell division [9]. The hits from this screen were then further studied to identify protein complexes [10]. Using gene tagging with green fluorescent protein (GFP) on bacterial artificial chromosomes (BAC) as part of the Mitocheck project, all the potential hits were localized in HeLa cells. Interestingly several proteins that were found to have a role in cell cycle regulation turned out to be localized on the Golgi apparatus. Among them, a protein whose function was unknown, C11ORF24, seemed particularly attractive. We named this protein C11ORF24. The mouse C11ORF24 tagged with GFP using the BAC system was localized on the Golgi apparatus [10].

Here we characterize the dynamics and transport of this protein. 

## Results and Discussion

### C11ORF24 is a type I transmembrane protein

**Figure 1:**
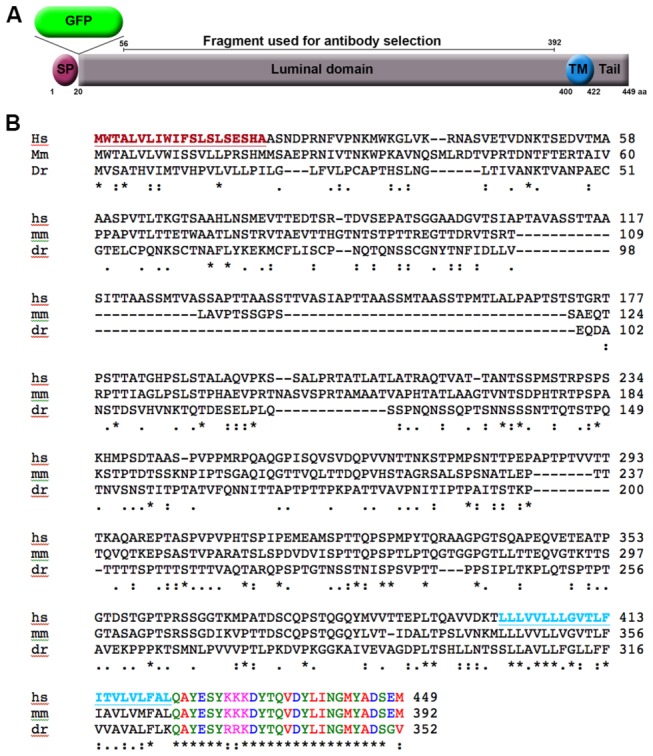
C11ORF24 sequence. (A) Putative domains of C11ORF24. SP: signal peptide, GFP: green fluorescent protein, TM: transmembrane domain, Tail: cytosolic tail. The numbers indicate the amino-acid position in the native protein. The putative signal peptide is underlined and colored in dark red and the putative transmembrane domain is underlined and colored in light blue. The tail domain is color-coded using Clustal W2 to highlight the similarities between different species. The position of the fragment used to raise the monoclonal antibody is indicated by the black bar. (B) The Homo sapiens (hs) C11ORF24 was aligned against its putative homologues in Mus musculus (mm, NP_082353.1) and *Dano* *rerio* (*Dr*, XP_690904.4) using Clustal W2 [13,14]. The cytosolic tail domain is well conserved in evolution.

In order to identify specific domains of C11ORF24, we analyzed the sequence of the protein. The C11ORF24 gene encodes a 449-amino acid protein. BLASTS, sequence motif and homology searches led us to the identification of three interesting domains: a signal peptide, a transmembrane domain and a cytosolic tail. The putative signal peptide was identified using a prediction software (http://bmbpcu36.leeds.ac.uk/prot_analysis/Signal.html, Figure 1A, SP). The putative transmembrane domain in the C-terminal region was detected using the HMMTOP prediction software [11,12] (Figure 1A, TM). The entire sequence of *Homo Sapiens (Hs*) C11ORF24 was aligned with its potential homologues in *Mus musculus (Mm*) and *Dano rerio* (*Dr*) using Clustal W2 [13,14] (Figure 1B). The N-terminal part of the protein showed little similarity between the different species. However, the tail region was highly conserved suggesting that it could play an important role in the function of C11ORF24.

### C11ORF24 is localized on the Golgi apparatus and the *trans*-Golgi network

**Figure 2:**
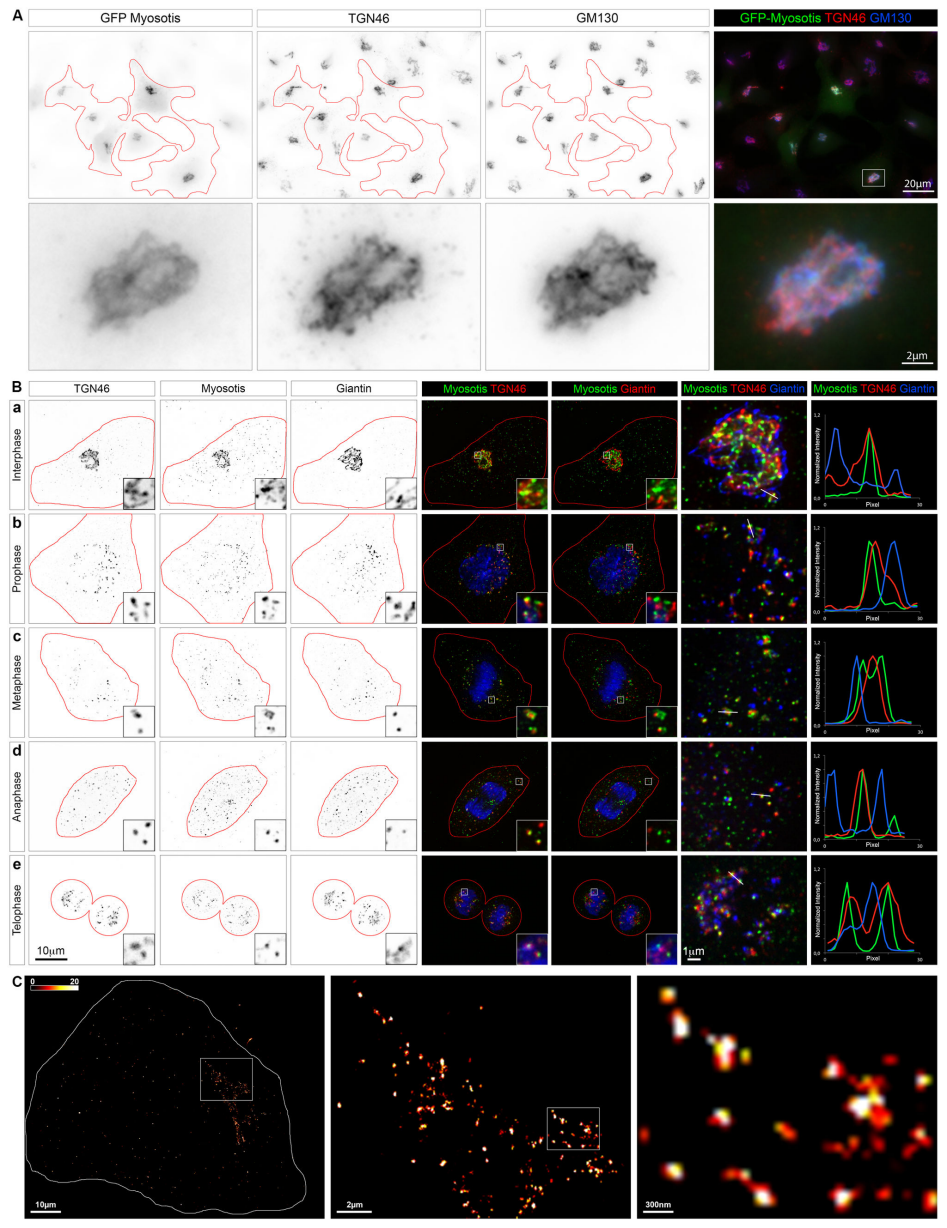
C11ORF24 is localized on the trans-Golgi Network. (A) HeLa cells were transfected with a construct encoding human C11ORF24 tagged with GFP and fixed 18 hours later. Cells were co-stained with TGN46 (middle, red) and GM130 (right, blue). The second line represents a closer view of the Golgi apparatus outlined by a rectangle in the merge image. (B) HeLa cells were fixed and co-stained with TGN46, C11ORF24 and Giantin. Colocalization is very strong between TGN46 and C11ORF24 (column 4) whereas it is lower with Giantin (column 5). Line profiles of the C11ORF24 (green), TGN46 (red) and Giantin (blue) fluorescence intensities from the lines in the magnified views are shown in column 7. The colocalization between TGN46 and C11ORF24 is clearly visible throughout mitosis (lines 2-5). (C) HeLa cells were stained for C11ORF24 and imaged using dSTORM super-resolution microscopy. A Fire LUT was used to facilitate visualization. A magnified view of the boxed region of the left image is shown in the middle and a magnified view of the boxed region of the middle image is shown on the right. The cell contour is outlined in red.

To investigate human C11ORF24 localization, we fused the human cDNA coding for C11ORF24 with a GFP tag. As expected from the sequence analysis, expression of the fusion protein with the GFP at the N-terminus was impossible and placing the GFP at the C-terminus greatly impaired protein expression (data not shown). For this reason, we inserted the GFP after the signal peptide (Figure 1A). The GFP-tagged C11ORF24 was expressed in HeLa cells for 18 hours. Cells were fixed and stained by immunofluorescence with antibodies against the TGN marker TGN46 and the *cis*-Golgi marker GM130. GFP-C11ORF24 is clearly localized on the Golgi apparatus, closely apposed to the two Golgi markers TGN46 and GM130 (Figure 2A). To assess the localization of the endogenous protein we raised a monoclonal antibody in mouse. The specificity of this antibody was confirmed by the loss of immunofluorescence staining after knockdown of C11ORF24 in HeLa cells (Figure S1). Immunofluorescence experiments in HeLa cells showed that the endogenous protein was closely apposed to the two Golgi markers Giantin and TGN46 therefore indicating that native C11ORF24 was localized on the Golgi apparatus (Figure 2B, a). Moreover, we confirmed that C11ORF24 behaved like other Golgi proteins upon Brefeldin A treatment: after 5 minutes of treatment C11ORF24 was present on tubes containing TGN46 (Figure S2, b) and upon longer treatment, the protein was present throughout the cell (Figure S2, c). C11ORF24 strongly colocalized with the TGN marker TGN46 as shown by the graph of fluorescence intensity along a line where C11ORF24 is in green and TGN46 is in red (Figure 2B, a). On the other hand, the *cis*-Golgi marker Giantin was closely apposed as shown by the blue line on the graph. It is also interesting to note that C11ORF24 staining was very punctate.

Since C11ORF24 was shown to be involved in cell cycle progression [9], we looked at the localization of the endogenous protein throughout mitosis (Figure 2B, b-e). From prophase to anaphase (Figure 2B, b-d), C11ORF24 was present on small punctate structures and was well co-localized with the TGN marker TGN46 whereas it was closely apposed to the *cis* marker Giantin (Figure 2B, b-d and line plot). In telophase when the Golgi apparatus forms a compact structure on both sides of the nucleus, C11ORF24 was still found on the TGN as shown by the co-localization with TGN46 (Figure 2B, e and line plot). To characterize the structures labeled with the anti-C11ORF24 antibody in more detail, we used an optical super-resolution fluorescence microscopy technique, direct stochastic optical reconstruction microscopy (dSTORM) [15-17] (Figure 2C). This technique allowed us to visualize the small sub-domains of the Golgi apparatus containing C11ORF24. Interestingly, TGN46 was also present on small punctate domains (data not shown). These results suggested that C11ORF24 was indeed present on the trans Golgi Network and may be sorted into specific sub-domains.

### C11ORF24 has a luminal domain and a small cytosolic tail

**Figure 3:**
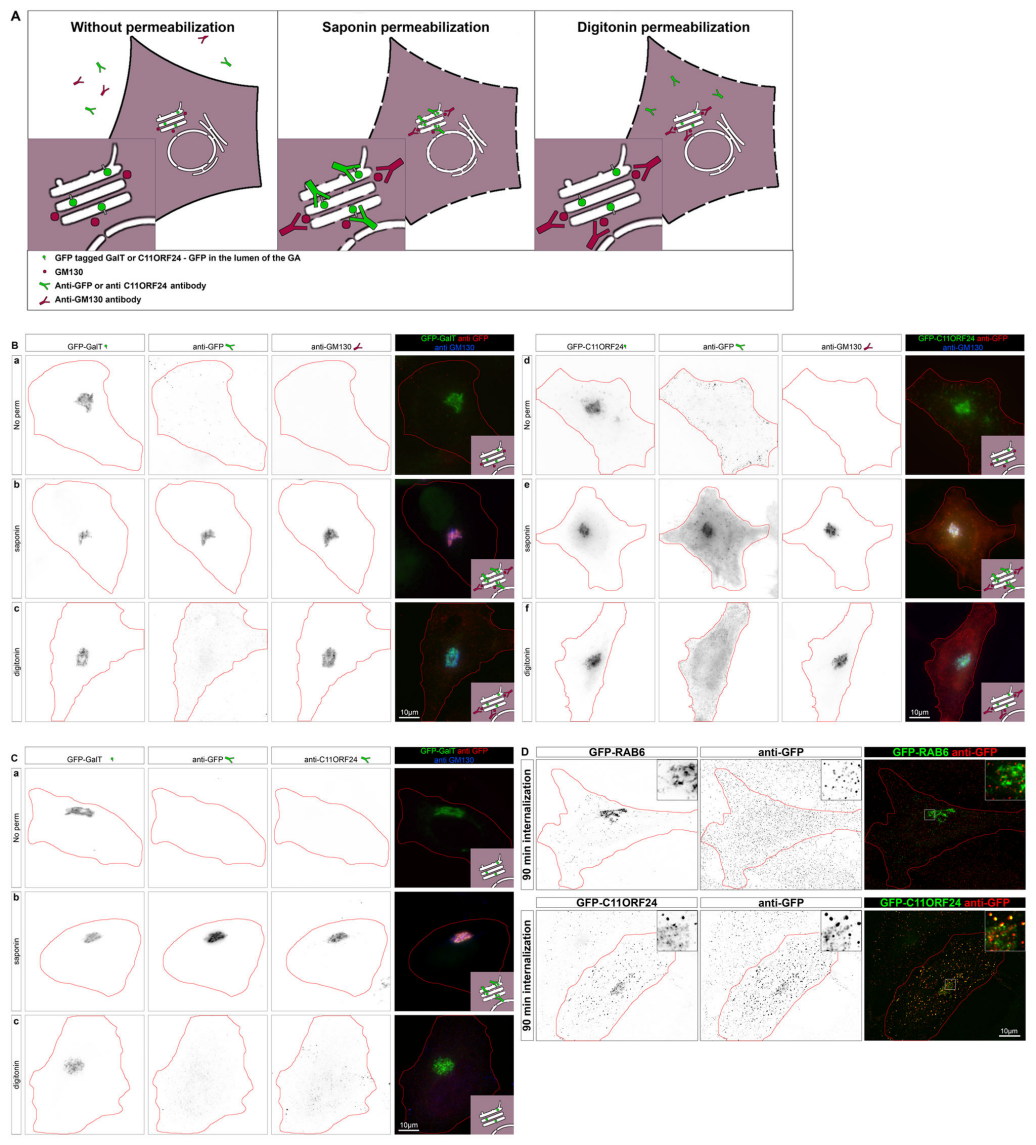
C11ORF24 has a long luminal domain and a short cytosolic tail. (A) Cartoon of a cell expressing GFP-tagged GalT or C11ORF24 and stained with an anti-GFP antibody and an anti-GM130 antibody. When the cells are not permeabilized (left) the antibodies don’t bind their targets. After saponin permeabilization (middle) all antibodies are able to reach their targets since all membranes are permeabilized. After digitonin permeabilization (right) only the cytosolic epitopes (GM130) are labeled whereas the luminal epitopes (GFP of GalT or C11ORF24) are protected by the intact Golgi membranes. Protocol modified from [18] (B) HeLa cells expressing GFP-GalT (a-c) or GFP-C11ORF24 (d-f) were stained with GFP and GM130 antibodies prior to fixation (a, d) or after fixation and permeabilization with saponin (b,e) or after fixation and permeabilization with digitonin (c,f). The GFP of C11ORF24 is accessible for the anti GFP antibody only after permeabilization of the Golgi membrane. (C) HeLa cells expressing GFP-GalT were stained with GFP and C11ORF24 antibodies prior to fixation (a) or after fixation and permeabilization with saponin (b) or after fixation and permeabilization with digitonin (c). The epitope of the C11ORF24 antibody is accessible only after permeabilization of the Golgi membrane. (D) HeLa cells were either transfected with GFP-RAB6 (line 1) or GFP-C11ORF24 (line 2) 18h prior to the incubation with the anti-GFP and internalization was performed at 37°C for 90 minutes. Cells were then fixed and stained with a secondary antibody and the localization of the anti-GFP antibody was compared with the GFP signal.

Sequence analysis suggested that C11ORF24 had a luminal domain and a short cytosolic tail. To test whether this topology was correct we used a modified version of the “Fluorescence Protease Protection” assay [18]. Our goal was to test whether the domain of C11ORF24 from the N-terminus to the transmembrane domain was indeed in the lumen of the Golgi apparatus. We took advantage of the fact that both the GFP tag and the epitope of the anti-C11ORF24 antibody were located in this region (Figure 1A). A cartoon of the experiment indicates the expected results (Figure 3A). As controls, we used GFP-tagged Galactosyltransferase (GalT), since the GFP of GalT is known to be in the lumen of the Golgi apparatus, and the peripheral protein of the Golgi apparatus GM130. The immunofluorescence was performed either without permeabilization (Figure 3A left), after saponin permeabilization (Figure 3A middle) or after digitonin permeabilization (Figure 3A right). To ensure that the cells were not permeabilized in the first condition, we performed immunofluorescence staining prior to fixation at 4°C. Without permeabilization none of the antibodies should be able to reach their targets on the Golgi apparatus (Figure 3A, left: anti-GFP or anti-C11ORF24 in green, anti GM130 in red). After saponin permeabilization all the membranes should be permeabilized allowing all antibodies to bind their epitopes (Figure 3A, middle: GFP of GalT or C11ORF24 and anti-C11ORF24 epitope are shown as a green dot inside the Golgi apparatus lumen, GM130 epitope is shown as a red dot outside of the Golgi apparatus). After digitonin permeabilization the plasma membrane should be permeabilized so the antibodies should be able to enter the cell however the Golgi membrane should not be permeabilized so the epitopes present in the lumen should not be accessible to antibodies (Figure 3A, right: GFP of GalT or C11ORF24 are not accessible whereas GM130 is accessible). As expected, in control cells expressing GFP-GalT none of the antibodies were able to reach their targets without permeabilization (Figure 3B, a), whereas after saponin permeabilization all the antibodies (anti-GFP and anti-GM130) targeted the Golgi apparatus (Figure 3B, b). After digitonin permeabilization the anti-GFP antibody did not detect the GFP of GalT since it was protected by the intact Golgi membrane. However the anti-GM130 antibody detected GM130 demonstrating that the plasma membrane was efficiently permeabilized (Figure 3B, c). In cells expressing GFP-C11ORF24 we observed the same result. Without permeabilization none of the antibodies could reach the Golgi apparatus (Figure 3B, d), after saponin permeabilization all the antibodies targeted the Golgi apparatus (Figure 3B, e), and, after digitonin permeabilization the anti-GFP antibody could not detect the GFP of C11ORF24, despite GM130 labeling showing that the plasma membrane was correctly permeabilized (Figure 3B, f). These results demonstrated that the N-terminal part of GFP-C11ORF24 was in the lumen of the Golgi apparatus. Using the anti-C11ORF24 antibody, we obtained the same result for the endogenous protein (Figure 3C). We concluded that C11ORF24 has a luminal domain in the Golgi apparatus.

**Figure 4:**
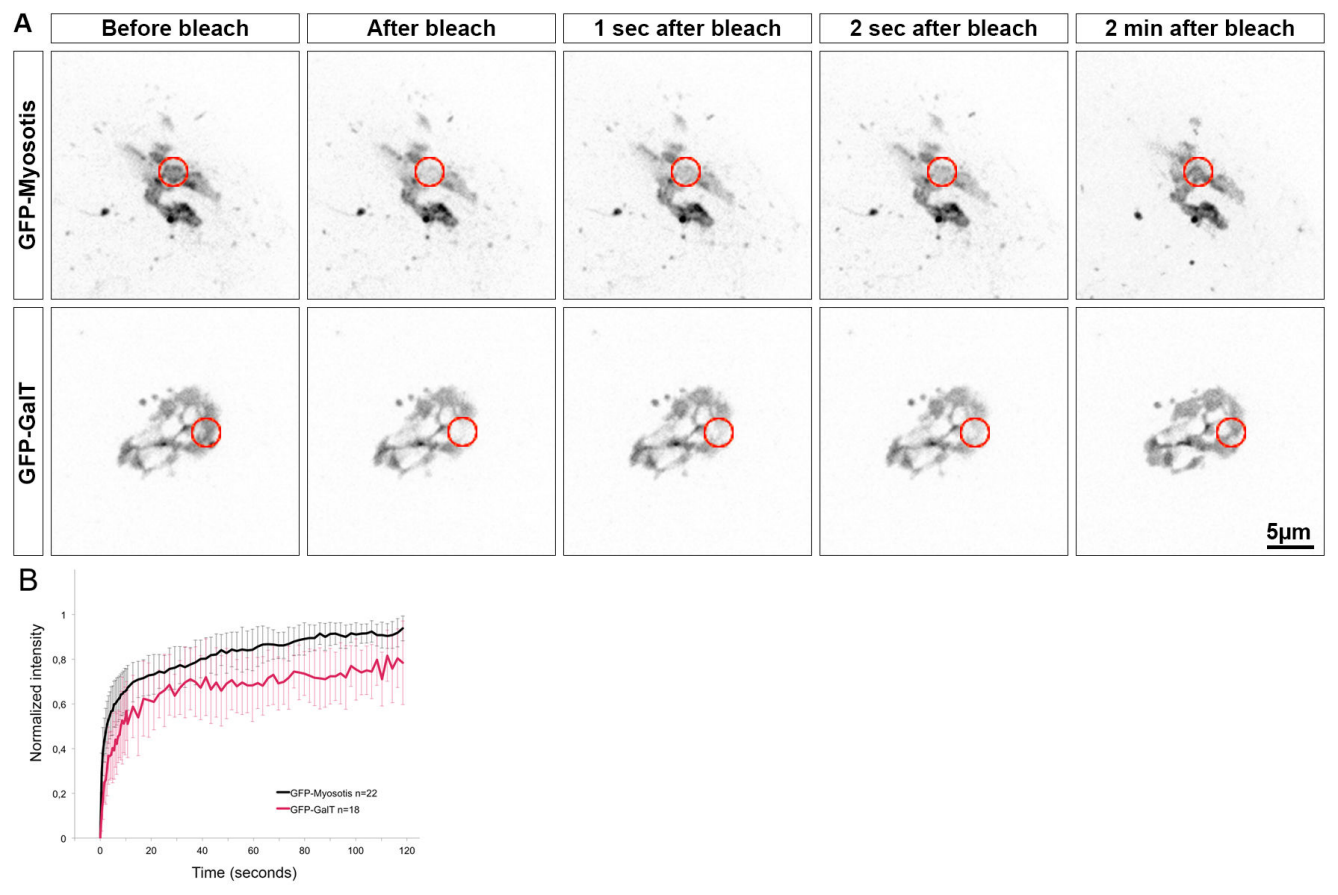
C11ORF24 is a transmembrane protein. (A) HeLa cells expressing GFP-C11ORF24 (line 1) or GFP-GalT (line 2) were bleached in a 3,5µm circular region of the Golgi apparatus (red circle) and then imaged for 2 minutes by spinning disk microscopy. The fluorescence recovery after photobleaching was measured in 3 independent experiments and plotted for GFP-GalT (red, n=18 cells) and GFP-C11ORF24 (black, n=22 cells). Error bars indicate the standard deviation. The recovery was similar for the two proteins.

To go further in the analysis of C11ORF24 dynamics in the Golgi membrane, we performed Fluorescence Recovery After Photo-bleaching (FRAP) on cells expressing GFP-tagged C11ORF24 or GFP-GalT as a control (Figure 4A shows one example). A small (3,5µm) region of the Golgi apparatus (Figure 4A, red circle) was photo-bleached and the recovery of fluorescence was recorded for 2 minutes in the same region. The images were corrected for photo bleaching due to the acquisition and the intensities were normalized.

The results obtained from three independent experiments are shown in the graph (Figure 4B, GFP-C11ORF24 in black and GFP-GalT in red). The dynamics of C11ORF24 in the Golgi membrane was similar to that observed for the transmembrane protein GalT in this study and in previous studies using other transmembrane Golgi enzymes [19,20]. Moreover this dynamic was slow compared to that of peripheral proteins of the Golgi apparatus likewise observed via photo-bleaching [21-23]. These results were in agreement with the sequence analysis and suggested that C11ORF24 was a transmembrane protein of the Golgi apparatus. 

### A pool of C11ORF24 is present at the plasma membrane and in endosomes

In order to understand the mechanism controlling C11ORF24 localization on the Golgi apparatus, we used an assay to perturb the pH gradient across organelle membranes as previously described [24]. This assay relies on the fact that the Golgi integral membrane protein GOLIM4 is localized on the Golgi apparatus at steady state but cycles to the plasma membrane and is transported back through the endosomes [24-26]. Upon monensin treatment GOLIM4 is blocked in endosomes. Cells were treated with monensin in the presence of cycloheximide for 1 hour and the localization of C11ORF24 was compared to the Golgi marker GM130. Without monensin treatment C11ORF24 was localized on the Golgi apparatus (Figure S2B, monensin 0min) whereas after 1 hour of treatment Golgi localization was lost and C11ORF24 was present on punctate structures (Figure S2B, monensin 60min). These results suggested that C11ORF24 was transported to the plasma membrane and recycled *via* the endosomes as is the case for GOLIM4 and TGN46 [24]. 

Since a pool of C11ORF24 is present at the plasma membrane (Figure S2B), its N-terminal domain should be exposed on the cell surface. To test this hypothesis we performed an antibody internalization assay. Cells expressing either GFP-RAB6 as a negative control protein that is not present on the cell surface or GFP-C11ORF24 were incubated with an anti-GFP antibody and internalization was followed at 37°C for 0, 15, 90 and 240 minutes (Figure 3D and Figure S3). As shown in Figure 3 and as expected, in the case of GFP-RAB6 the anti-GFP antibody was not internalized (Figure 3D and Figure S3, GFP-RAB6). On the contrary, for GFP-C11ORF24, the anti-GFP antibody was internalized. Indeed, the antibody was first bound to the plasma membrane (Figure S3, 0 min.), then internalized into endosomes (Figure S3, 15 min.) and finally transported to the Golgi apparatus (Figure 3, 90 min. and Figure S3, 240 min.). These results thus confirmed that C11ORF24 was present at the plasma membrane and that its N-terminal domain was at the cell surface.

It is interesting to note that internalization of the endogenous protein was not visible (data not shown). This could be due to the very low expression level of the protein: if only a small fraction of the protein is present at the plasma membrane, the antibody fixed on the plasma membrane will not be detected. The plasma membrane localization of C11ORF24 was not a consequence of the presence of the GFP-tag since similar results were obtained with a non-tagged version of the protein (data not shown), nor was it due to protein overexpression since the cells used in these assays show a very low expression level.

### C11ORF24 is present on RAB6 positive transport carriers

**Figure 5:**
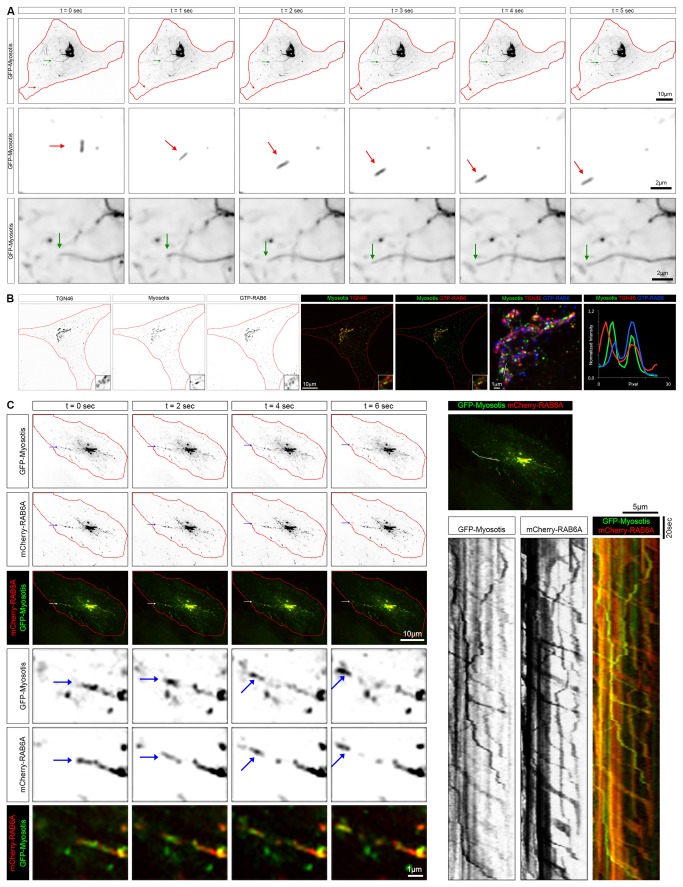
C11ORF24 is present on RAB6 positive transport carriers. (A) HeLa cells expressing GFP-C11ORF24 were imaged every second for 5 minutes by spinning disk microscopy. The red arrow indicates a small transport carrier whereas the green arrow point to a long tube connected to the Golgi apparatus. A magnified view of these structures is shown in line 2 and 3. (B) HeLa cells were fixed and co-stained with TGN46, C11ORF24 and GTP-RAB6. Colocalization is very strong between TGN46, C11ORF24 and GTP-RAB6. A line profile of the C11ORF24 (green), TGN46 (red) and GTP-RAB6 (blue) fluorescence intensities from the lines in the magnified views are shown in column 7. (C) HeLa cells expressing GFP-C11ORF24 and mCherry-RAB6 were imaged every second for 5 minutes by spinning disk microscopy. The arrow indicates a transport carrier positive for GFP-C11ORF24 (green) and for mCherry-RAB6 (red). A magnified view of this structure is shown lines 4-6. (D) Transport carriers were visualized using a kymograph that was made along the line drawn on the merge image. All the dynamic elements positive for GFP-C11ORF24 (left and merge green) were positive for mCherry-RAB6 (middle and merge red).

To investigate the dynamics of C11ORF24 we monitored the localization of GFP-tagged C11ORF24 in HeLa cells by spinning disk confocal microscopy (Figure 5A). GFP- C11ORF24 was present on the Golgi apparatus and on transport carriers like tubulo-vesicular structures (Figure 5A, red arrow a and b) and tubes (Figure 5A, green arrow a and c). These structures are highly dynamic over time (Figure 5A time 0 to 5 seconds and movie S1). The localization and dynamics of C11ORF24 mirrored that of GFP-tagged RAB6A [27]. RAB6, one of the most conserved RAB proteins in evolution, is localized on the membranes of the Golgi apparatus and the *trans-*Golgi network [28,29]. Several studies from our lab and others have revealed that RAB6 regulates various trafficking pathways both in the anterograde and retrograde routes at the level of the Golgi apparatus and the TGN [30-34].

In order to determine whether C11ORF24 was present on RAB6 positive structures, we labeled HeLa cells with antibodies against C11ORF24, TGN46 and GTP-RAB6 (Figure 5B). C11ORF24 colocalized with RAB6 as shown on the magnified view of the Golgi apparatus and on the line profile of the C11ORF24, TGN46 and RAB6 fluorescence intensities. We therefore tested whether the two proteins colocalized on the same transport carriers. HeLa cells expressing both GFP-C11ORF24 and mCherry-RAB6A were followed using spinning disk microscopy (Figure 5C, time 0 to 6 seconds from left to right and movie S2). We found that RAB6 was present on all dynamic C11ORF24 positive structures. One example is shown in the magnified view of a transport carrier indicated by the arrow (Figure 5C, lines e-f). The colocalization was also clearly visible on the kymograph (Figure 5D) corresponding to the line drawn on the top image. All dynamic structures positive for C11ORF24 (left) were also positive for RAB6 (middle). 

These results suggested that C11ORF24 could either be involved in the RAB6 dependent transport or that C11ORF24 could be transported through RAB6 positive carriers.

### C11ORF24 is not necessary for the transport of classical cargos of the RAB6 pathway

**Figure 6:**
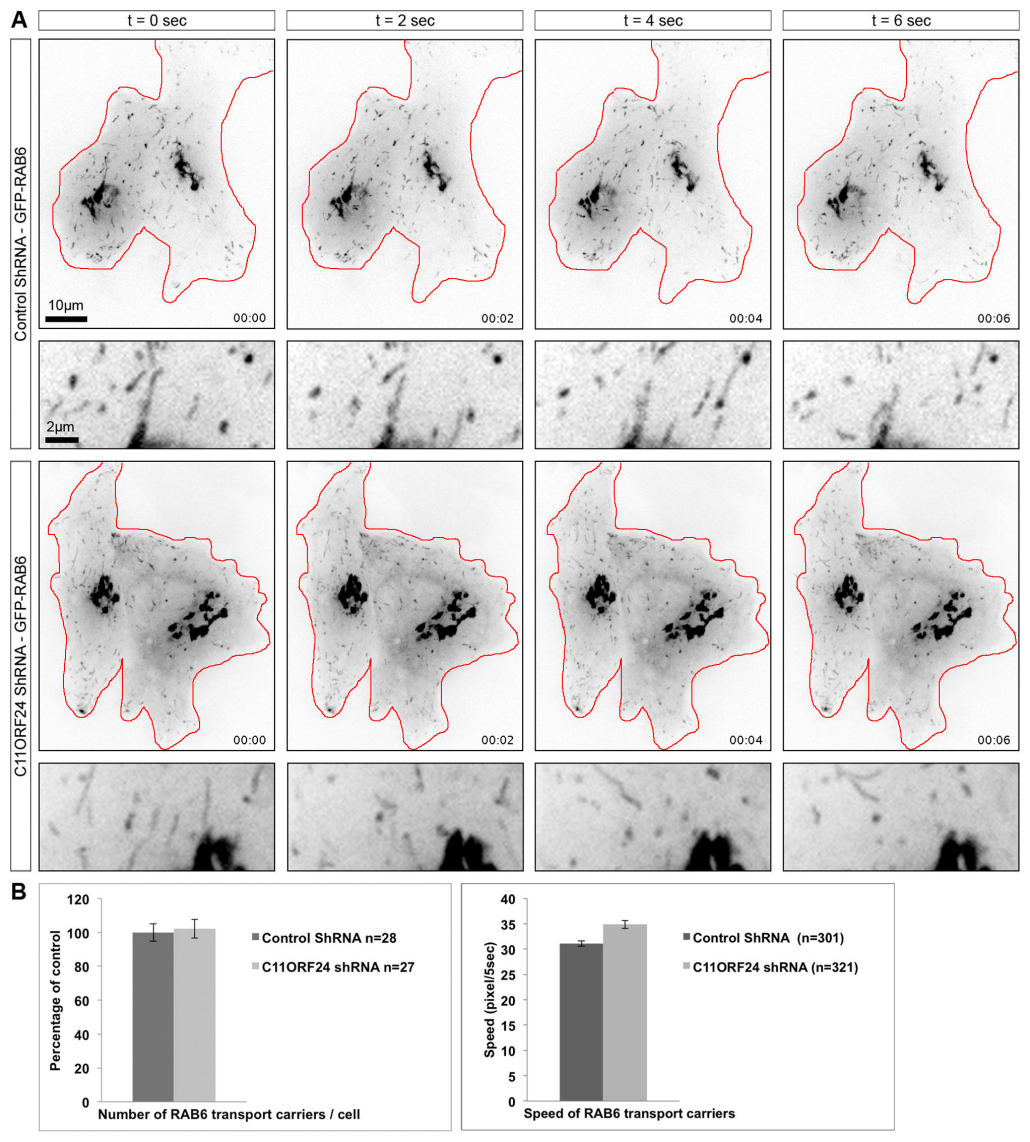
C11ORF24 is not necessary for the formation of RAB6 positive transport carriers. (A) After a 48 hours knockdown by shRNA HeLa cells were transfected with GFP-RAB6 and the next day they were imaged every second for 30 seconds by spinning disk microscopy. Snap shots from the Movies S1 and S2 are presented for the control shRNA (upper lines) compared to the C11ORF24 shRNA (lower lines). (B) The number of RAB6 positive transport carrier per cell and the speed of theses carriers was then quantified for both treatments from 3 independent experiments and expressed as a percentage of the control. The number of cells is indicated for each treatment on the graphs (n). The error bars represent the SEM.

In order to determine whether C11ORF24 plays a role in RAB6 dependent transport, we followed RAB6 dynamics after C11ORF24 knockdown. HeLa cells stably expressing an inducible control or C11ORF24 shRNA were treated for 48 hours with doxycycline to obtain a complete knockdown of C11ORF24. Cells were then transfected with GFP-RAB6 and observed using spinning disk microscopy (Figure 6A, movie S3 for control shRNA and movie S4 for C11ORF24 shRNA). In both control and knockdown cells, we observed RAB6-positive transport carriers moving throughout the cell. The number and speed of transport carriers was then quantified in the control and in C11ORF24 knockdown cells (Figure 6B). After shRNA treatment, the number and distribution of RAB6 transport carriers was not affected. We only observed a small increase in the speed of RAB6 transport carriers. We concluded that C11ORF24 had no function in the formation and movement of RAB6 transport carriers. 

We then tested whether C11ORF24 was necessary for RAB6 dependent transport of several cargos. After a 48-hours knockdown of C11ORF24, cells were incubated for 1 hour with Alexa488 coupled Shiga toxin B fragment (STxB) at 4°C. After changing the medium cells were transferred to 37°C and fixed after 0, 15, 90 or 240 minutes. The localization of STxB was then followed at each time point. No difference was observed between the cells treated with the control or the C11ORF24 shRNA (Figure S4A). These results suggested that C11ORF24 was not involved in the internalization of Shiga toxin. We also tested the effect of knockdown of C11ORF24 on the secretion of ^tsO45^VSVG. We followed the secretion of GFP-^tsO45^VSVG in cells depleted for C11ORF24 compared to the control shRNA. Cells were incubated overnight at 40°C to retain ^tsO45^VSVG in the endoplasmic reticulum and secretion was assessed after 0, 30, 120 minutes at 32°C. No defects in the secretion of ^tsO45^VSVG were observed (Figure S4B). These findings suggested that C11ORF24 was not required for RAB6 function and could instead be transported through RAB6 positive carriers. However we cannot exclude another possibility, namely that C11ORF24 could be involved in a different trafficking route that we have not tested.

### C11ORF24 is not involved in cell cycle regulation

Since C11ORF24 was proposed to be involved in cell cycle regulation [9], we studied the effect of C11ORF24 knockdown on mitosis. Knockdown was achieved similarly to what was described in the transport assay. Cells were then observed for 3 days using phase contrast on a video-microscope (Figure S5A). Representative images are shown for the control shRNA and for the C11ORF24 shRNA at the first time point (t=0) and after 2 days of observation (t=48h). Proliferation was quantified for the two cell lines (Figure S4B, 48h Proliferation). Cell number increases about 4 fold in 48h regardless of C11ORF24 knockdown. In order to determine whether there was any delay in a specific step of mitosis, cells were fixed and stained using a Phospho-histone antibody to visualize the early steps of mitosis and DAPI to visualize DNA. The number of cells positive for P-histone (Figure S5B, P-histone), of cells in metaphase (Figure S5B, Metaphase) or in anaphase (Figure S5B, Anaphase) was quantified in the two cell lines. We observed that about 4% of the cells were positive for P-histone, about 2,5% were in metaphase and about 0,5% were in anaphase. No significant difference was observed between the control cells and the cells where C11ORF24 was knocked down. We were not able to reproduce the mitotic phenotype described in the esiRNA based genome wide screen [9] (Figure S5). This phenotype could be the result of an off-target effect of the esiRNA since it is not rescued by expression of an esiRNA resistant construct of C11ORF24. The other possibility is that our knockdown was not efficient enough. The anti-C11ORF24 antibody did not detect any specific bands by western blot, and therefore we could not use this method to quantify the efficiency of our knockdown. However, by immunofluorescence we observed a complete loss of C11ORF24 signal on the Golgi apparatus. It is therefore likely that C11ORF24 does not play a key role in mitosis.

In conclusion here we report that the human C11ORF24 is localized on the TGN and on transport carriers positive for RAB6, although C11ORF24 is not involved in RAB6 dependent transport. We also shown that C11ORF24 has a signal peptide and is a type I transmembrane protein with a luminal domain and a short cytosolic tail that is well conserved in evolution, but that C11ORF24 does not appear to play a role in the Golgi mitotic checkpoint.

## Material and Methods

### Identification of C11ORF24 domains

The signal peptide was identified using the prediction software: http://bmbpcu36.leeds.ac.uk/prot_analysis/Signal.html. The transmembrane domain was identified as previously described [11,12] from this address: http://www.enzim.hu/hmmtop/index.php. Human C11ORF24 sequence was aligned with its homologues from different species using Clustal omega as previously described [14,35].

### GFP-C11ORF24 construct

The human C11ORF24 cDNA was obtained from Origene (No.: SC122931 Homo sapiens chromosome 11 open reading frame 24 DNA NM_022338.2). The full-length cDNA was amplified using the primers FL_for, 5’CGTATCGCTAGCATGTGGACAGCTCTTGTG and FL_rev, 5’GTGGCGACCGGTGGCATTTCTGAGTCCGCATA and C11ORF24 was inserted in the clontech vector pEGFP N1 in the NheI and AgeI sites (pEGFP-N1-C11ORF24). The cDNA sequence lacking the signal peptide was then amplified using the primers noSP_for, 5’TCAGTCTGTACAGCGCATCCAACGATCCACGC and noSP_rev, 5’GTCGAGGATCCTCACATTTCTGAGTCCGCATA and the fragment was inserted in the clontech vector pEGFP C1 between BsrGI and BamHI (pEGFP-C1-noSP-C11ORF24). The signal peptide was amplified by PCR from the pEGFP-N1-C11ORF24 using the primers SP-for, 5’TGTATCATATGCCAAGTACGC and SP_rev, 5’TAGCTAACCGGTCCCGCATGGCTTTCAGATAAGG and inserted before the GFP in the pEGFP-C1-noSP-C11ORF24 using the NdeI and AgeI sites (pEGFP-C1-C11ORF24). shRNA constructs in the inducible lentiviral system pTRIPZ were obtained from Openbiosystem for the control non-silencing shRNA (# RHS4743) and the C11ORF24 shRNA (#RHS4696-200755138, Mature Sense for V3THS_341691 TGGAAACAGTTGATAATAA).

### Cell culture, transfection and cell lines

HeLa cells were grown in DMEM medium (Gibco) supplemented with 10% fetal bovine serum at 37°C in a 5% CO2 humidified incubator. For the expression of GFP-C11ORF24, HeLa cells grown in 6 well plates were transfected using Xtrem9 (Roche) following the manufacturer’s instructions 18 hours prior to observation. For the knockdown of C11ORF24, lentiviral vectors encoding the different shRNA (control or C11ORF24) were produced by cotransfection of the VSV-G plasmid and the packaging psPax2 plasmid in 293T cells [36] and HeLa cells were transduced with the supernatant from these cells. Knockdown was complete after induction of shRNA expression for 48h by a doxycycline treatment (1µg/mL). HeLa cells stably expressing GFP-tagged GalT were generated as a part of a BAC TransgeneOmics project and described previously [37].

### Immunofluorescence

The cells were fixed with either 100% methanol at -20°C for 4 min or 3% paraformaldehyde (PFA) at room temperature for 30 min, washed with PBS, incubated with PBS-0.1 M NH4Cl for 5 min, washed again with PBS and permeabilized with 0.05% saponin for 20 min. Primary antibodies: anti-Giantin hFc TA10 1/50, sheep anti-TGN46 (AbD Serotec, 1/1000), mouse anti-GFP (Roche, 1/400), mouse anti-GM130 (Transduction Laboratories, 1/1000), DAPI 1/1000. The anti-C11ORF24 antibody was raised in mouse. A long soluble fragment of the protein (Figure 1A) tagged with glutathione S transferase (GST) was expressed in bacteria and purified. This fragment was then injected in mice and the antibodies obtained from cell supernatants were purified against a maltose binding protein (MBP) tagged version of the same fragment of the protein. After selection of the best hybridoma it was sent for sequencing to SydLabs (http://www.sydlabs.com/) and the heavy and light chains were subcloned and fused to the human Fc as previously described [38]. Secondary antibodies: anti-human Cy3, anti-mouse Cy3, anti-mouse Alexa488, anti-sheep Cy5, anti-mouse Alexa647 (Jackson laboratories, 1/400). Fixed cells were visualized using a Nikon Eclipse 80i Upright microscope with a CoolSnapHQ2 camera (Photometrics, 100x objective CFI Plan Apo VC NA 1.4 WD 0.13 DIC Oil for 3D acquisitions), or with a Leica DMRA and a CoolSnapHQ2 camera, 100x objective NA 1.25 oil pH 3 CS (HCX PL APO), and Metamorph. 3D stacks were acquired and deconvolved to build a projection on one plane on Metamorph. Line profiles of the fluorescence intensities were done using Fiji (http://fiji.sc/) and the kymographs were made using the Kymograph plugin (http://www.embl.de/eamnet/html/body_kymograph.html). 

### Direct Stochastic Optical Reconstruction Microscopy (dSTORM)

Samples were imaged at room temperature in a closed chamber (Ludin Chamber, Life Imaging Services, Switzerland) mounted on an inverted motorized microscope (Nikon Ti, Japan) equipped with a 100X 1.45NA PL-APO objective and a perfect focus system, allowing long acquisition in oblique illumination mode (Roper, France). Imaging was performed in an extracellular solution containing reducing and oxygen scavenging system, according to the dSTORM protocol [15-17]. At the beginning of the experiment, the ensemble fluorescence of Alexa Fluor 647 was first converted in to dark state using a 640nm laser (Coherent, USA) at 30-50 kW/cm^2^ intensity. Once the ensemble fluorescence was converted into the desired density of single molecules per frame, the laser power was reduced to 7-15 kW/cm^2^ and imaged continuously at 20FPS (50ms exposure time) for 20,000 frames. The number of single molecules detected per frame was controlled using a 405 nm laser (Omicron, Germany). The laser powers were adjusted to keep a specific level of stochastically activated molecules, which were well separated during the acquisition. Both the ensemble and single molecule fluorescence was collected by the combination of a dichroic and emission filter (D101-R561 and F39-617 respectively, Chroma, USA and quad-band dichroic filter (Di01-R405/488/561/635,Semrock, USA). The fluorescence was collected using a sensitive 512x512 EM-CCD (Evolve, Photometric, USA). 3D localization was performed using the N-STORM astigmatic lens (Nikon) located in front of the CCD camera. Single molecule localization and reconstruction was performed online using automatic feedback control on the lasers, enabling optimal molecule density during the acquisition [39,40]. The acquisition sequence was driven by Metamorph software (Molecular Devices, USA) in streaming mode using an area equal to or less than 256x256 pixel as region of interest. We used multicolor fluorescent microbeads (Tetraspeck, Invitrogen) to register long-term acquisitions and to correct for lateral drifts and chromatic shifts. A spatial resolution of 14 nm was measured using centroid determination on 100 nm Tetraspeck beads acquired with similar signal to noise ratio than single-molecule images.

### Topology of C11ORF24

For the immunofluorescence without permeabilization cells were incubated with the antibody at 4°C then fixed by addition of paraformaldehyde 3% in the medium and stained with the secondary antibody in PBS. For the saponin treatment cells were processes as previously described in the immunofluorescence section. Finally, for the digitonin treatment cells were fixed and permeabilized using 0,8µM digitonin for 20 minutes at room temperature. The immunofluorescence was then performed in the presence of 0,8µM digitonin.

### Antibody internalization

HeLa cells were transfected with GFP-RAB6 or GFP-C11ORF24 using Xtrem9 (Roche) following the manufacturer’s instructions 18 hours prior to observation. Cells were then incubated at 4°C with the anti-GFP (Roche, 1/400) for 1 hour. After a 0, 15, 90, 240 minutes incubation at 37°C cells were processed for immunofluorescence.

### Drug treatments

Brefedlin A treatment was performed as described previously [41]. Briefly cells were incubated with the drug for 0, 5, 60 minutes at 37°C and were processed for immunofluorescence.

Monensin treatment was performed as described previously[24]. Cells were treated with cycloheximide for 30 minutes and then incubated with monensin for 0 or 60 minutes and then processed for immunofluorescence.

### Retrograde transport of STxB

Retrograde transport of Alexa488-labelled STxB to the endoplasmic reticulum was performed as described previously [42] after 48 hours of shRNA induction. HeLa cells were incubated with Alexa488-STxB for 1 hour at 4°C. After changing the medium, cells were transferred at 37°C for 15, 90 or 240 minutes. Cells were then fixed and processed for immunofluorescence.

### Secretion of ^tsO45^VSVG

Secretion of GFP-^tsO45^VSVG from the endoplasmic reticulum to the plasma membrane was performed as described previously [43]. After 48 hours of shRNA induction, cells were transfected with GFP-^tsO45^VSVG using Xtrem9 (Roche) following the manufacturer’s instructions 24 hours prior to observation. Cells were incubated overnight at 40°C to retain the protein in the endoplamic reticulum and then switched to 32°C for 0, 30, 120 minutes to follow the secretion.

### Live cell imaging

For the GFP-C11ORF24 or GFP-C11ORF24 with mCherry-RAB6A movies or GFP-RAB6 after a 48h induction of shRNA, HeLa cells cultured in glass bottom Fluorodish cell culture dishes (World Precision Instruments) were transfected using Xtrem9 (Roche) following the manufacturer’s instructions 18 hours prior to observation. The images were acquired on a spinning disk microscope. The Spinning disk microscope is based on a CSU-X1 Yokogawa head mounted on an inverted Ti-E Nikon microscope equipped with a motorized XY Stage. Images were acquired through a x100 1.4NA Plan-Apo objective with a Photometrics Coolsnap HQ2 CCD camera. Optical sectioning was achieved using a piezo stage (Mad City Lab). A Roper/ Errol laser lounge is equipped with 491 and 561 nm laser diodes, delivering 50 mW each, coupled to the spinning disk head through a single fiber. Multi-dimensional acquisitions are performed in streaming mode using Metamorph 7.7.6 software. Images are collected every second (500msec exposure). The data shown in the movies are obtained using ND-SAFIR (N-Dimensional – Structure Adaptive Filtering for Image Restoration) # INRIA/INRA 2007 as previously described [44] [45]. The number and the speed of RAB6 transport carriers was measured using the ImageJ software.

For FRAP analyses with GFP-GalT, GFP-C11ORF24, HeLa cells were maintained in culture medium in glass bottom Fluorodish cell culture dishes and imaged on a similar spinning disk microscope equipped with FRAP head (Errol and Roper), 18 h after transfection. Images were collected before bleaching of the 3,5µm region of the Golgi apparatus and after photo-bleaching images were acquired every 500 ms for 5 seconds and every 2 seconds for 2 minutes. Images were processed using Metamorph software. After correction of the photo-bleaching due to acquisition, the background was subtracted. The intensity of fluorescence was then normalized and plotted on the graph.

## Supporting Information

Figure S1 **Antibody specificity and shRNA efficiency.** HeLa cells were transiently transfected with an inducible control or C11ORF24 shRNA and treated with doxycycline to induce the expression of the shRNA. After 48 hours of induction cells were fixed and stained with an anti-C11ORF24 antibody.
(TIF)

Figure S2 **C11ORF24 localization after Brefeldin A treatment.** (A) HeLa cells were either untreated (Control), treated with Brefeldin A for 5 minutes or treated with Brefeldin A for 60 minutes. Cells were then fixed and stained with anti-C11ORF24 (green) and anti-TGN46 (red) antibodies. (B) HeLa cells were treated with cycloheximide and then either directly processed (monensin 0 min) or treated with monensin for 60 minutes. Cells were then fixed and stained with anti-C11ORF24 (green) and anti-GM130 (red) antibodies.
(TIF)

Figure S3 **C11ORF24 is present at the plasma membrane and is internalized.** HeLa cells were either transfected with GFP-RAB6 (left pannel) or GFP-C11ORF24 (right pannel) 18h prior to the incubation with the anti-GFP antibody and internalization was performed at 37°C for 0, 15 and 240 minutes. Cells were then fixed and stained with a secondary antibody and the localization of the anti-GFP antibody (red) was compared with the GFP signal (green).
(TIF)

Figure S4 **C11ORF24 is not necessary for the transport of classical cargos of the RAB6 pathway.** (A) HeLa cells stably expressing either an inducible control shRNA or an shRNA against C11ORF24 were treated with doxycycline for 48 hours to induce shRNA and RFP expression. Cells were then incubated with STxB-488 for one hour at 4°C. Internalization was then performed at 37°C for the indicated times. Finally cells were fixed and stained with an anti-C11ORF24 antibody and the localization of STxB-488 over time was observed. (B) After a 48 hours knockdown cells were transfected with GFP-^tsO45^VSVG and incubated overnight at 40°C to retain the protein in the endoplamic reticulum and then switched to 32°C for 0, 30, 120 minutes to follow the secretion. Cells were then fixed and stained with and anti-VSVG antibody without permeabilization to detect specifically the arrival of the protein at the plasma membrane.
(TIF)

Figure S5 **C11ORF24 is not necessary for cell cycle progression.** HeLa cells stably expressing either an inducible control shRNA or an shRNA against C11ORF24 were treated with doxycycline for 48 hours to induce shRNA and RFP expression. A) Cells were then plated on microscopy chambers in the presence of doxycycline and observed every 10 minutes for 3 days by phase contrast. A snap shot of one representatives field is shown and the quantification is shown in B. B) Cells were fixed and stained with a phospho-histone antibody (P-Histone graph), DAPI to detect the DNA (Metaphase and Anaphase graphs), or counted during the movie shown in A (48h proliferation graph).
(TIF)

Movie S1 **GFP-C11ORF24 dynamics.** HeLa cells expressing GFP-C11ORF24 were imaged every second for 5 minutes by spinning disk microscopy. Small transport carrier and long tubes connected to the Golgi apparatus or not connected in the periphery of the cell are visible. Snap shots of the movie are presented in Figure 5 A.
(MOV)

Movie S2 **GFP-C11ORF24 and mCherry-RAB6 colocalization.** HeLa cells expressing GFP-C11ORF24 and mCherry-RAB6 were imaged every second for 5 minutes by spinning disk microscopy. Snap shots of the movie are presented in Figure 5 C.
(MOV)

Movie S3 **GFP-RAB6 dynamics after C11ORF24 knockdown.** After a 48 hours knockdown with control shRNA, HeLa cells were transfected with GFP-RAB6 and imaged every second for 30 seconds by spinning disk microscopy. Snap shots of the movie are presented in Figure 6.
(MOV)

Movie S4 **GFP-RAB6 dynamics after C11ORF24 knockdown.** After a 48 hours knockdown with C11ORF24 shRNA, HeLa cells were transfected with GFP-RAB6 and imaged every second for 30 seconds by spinning disk microscopy. Snap shots of the movie are presented in Figure 6.
(MOV)
