# Supplementary figures and images for: C11ORF24 Is a Novel Type I Membrane Protein That Cycles between the Golgi Apparatus and the Plasma Membrane in Rab6-Positive Vesicles
Source: PLoS One. 2013 Dec 2;8(12):e82223. doi: 10.1371/journal.pone.0082223 (PMC3846831; doi:10.1371/journal.pone.0082223)

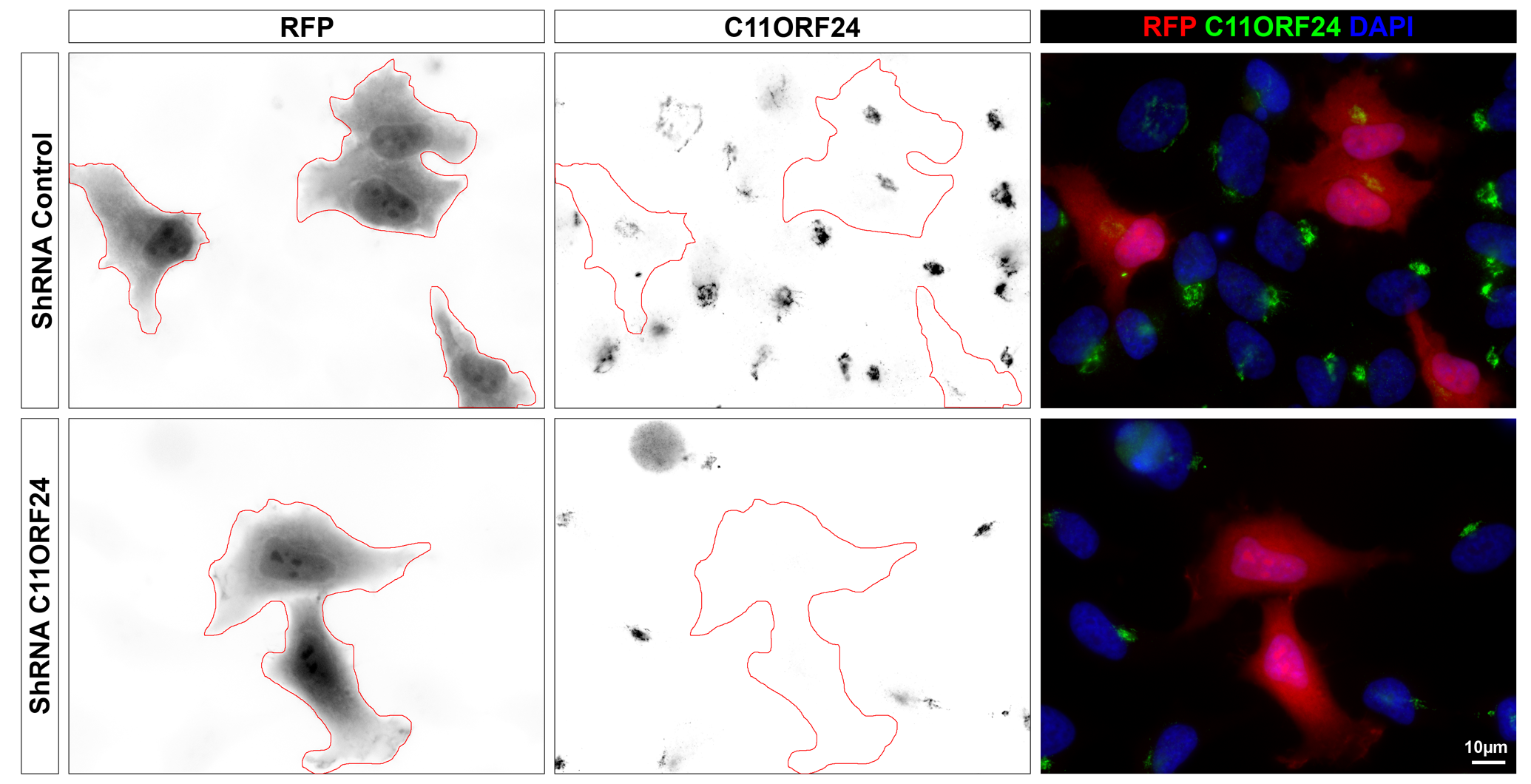

Supplement: Figure S1 — Antibody specificity and shRNA efficiency. HeLa cells were transiently transfected with an inducible control or C11ORF24 shRNA and treated with doxycycline to induce the expression of the shRNA. After 48 hours of induction cells were fixed and stained with an anti-C11ORF24 antibody. (TIF) [file pone.0082223.s001.tif]

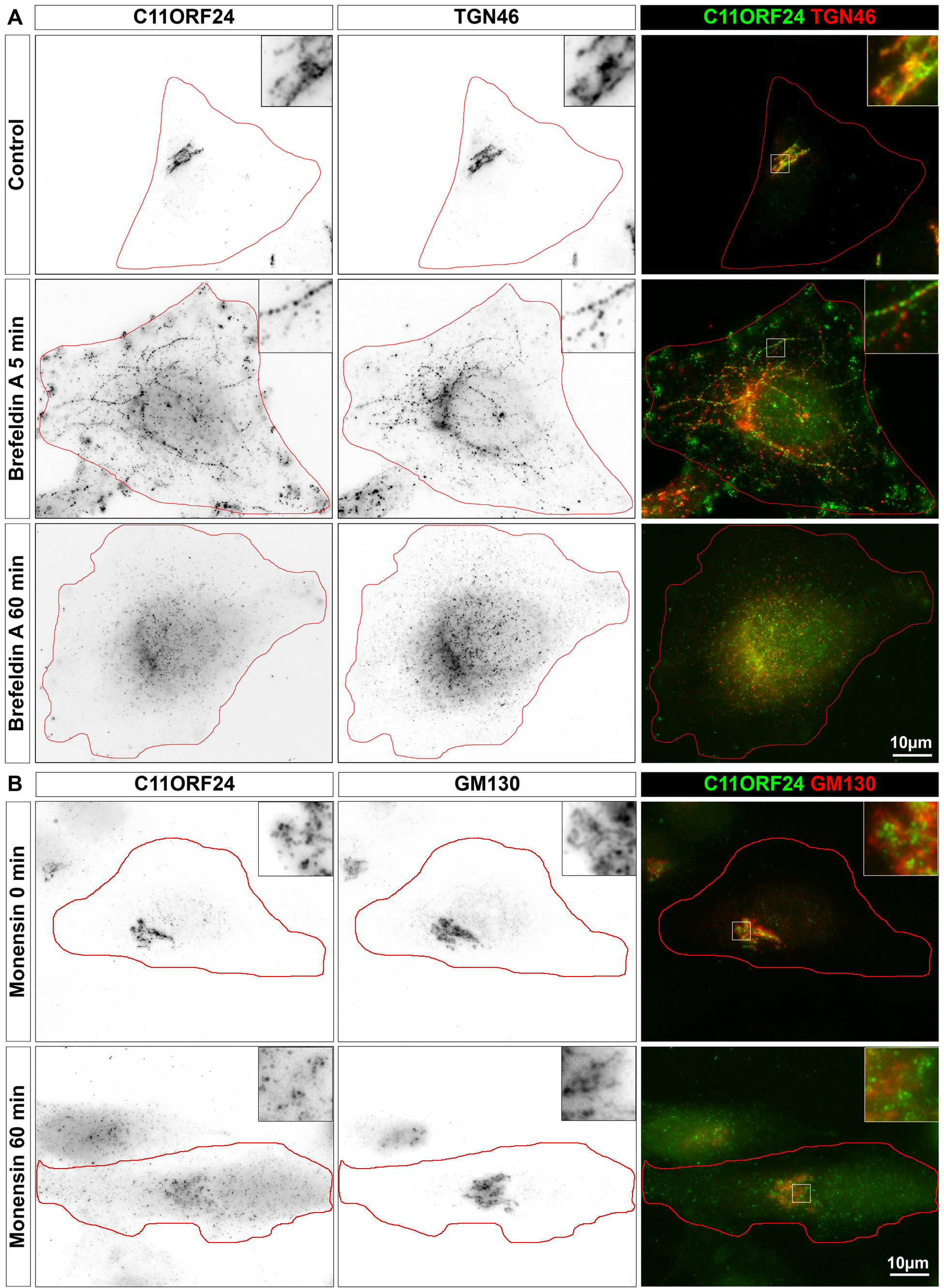

Supplement: Figure S2 — C11ORF24 localization after Brefeldin A treatment. (A) HeLa cells were either untreated (Control), treated with Brefeldin A for 5 minutes or treated with Brefeldin A for 60 minutes. Cells were then fixed and stained with anti-C11ORF24 (green) and anti-TGN46 (red) antibodies. (B) HeLa cells were treated with cycloheximide and then either directly processed (monensin 0 min) or treated with monensin for 60 minutes. Cells were then fixed and stained with anti-C11ORF24 (green) and anti-GM130 (red) antibodies. (TIF) [file pone.0082223.s002.tif]

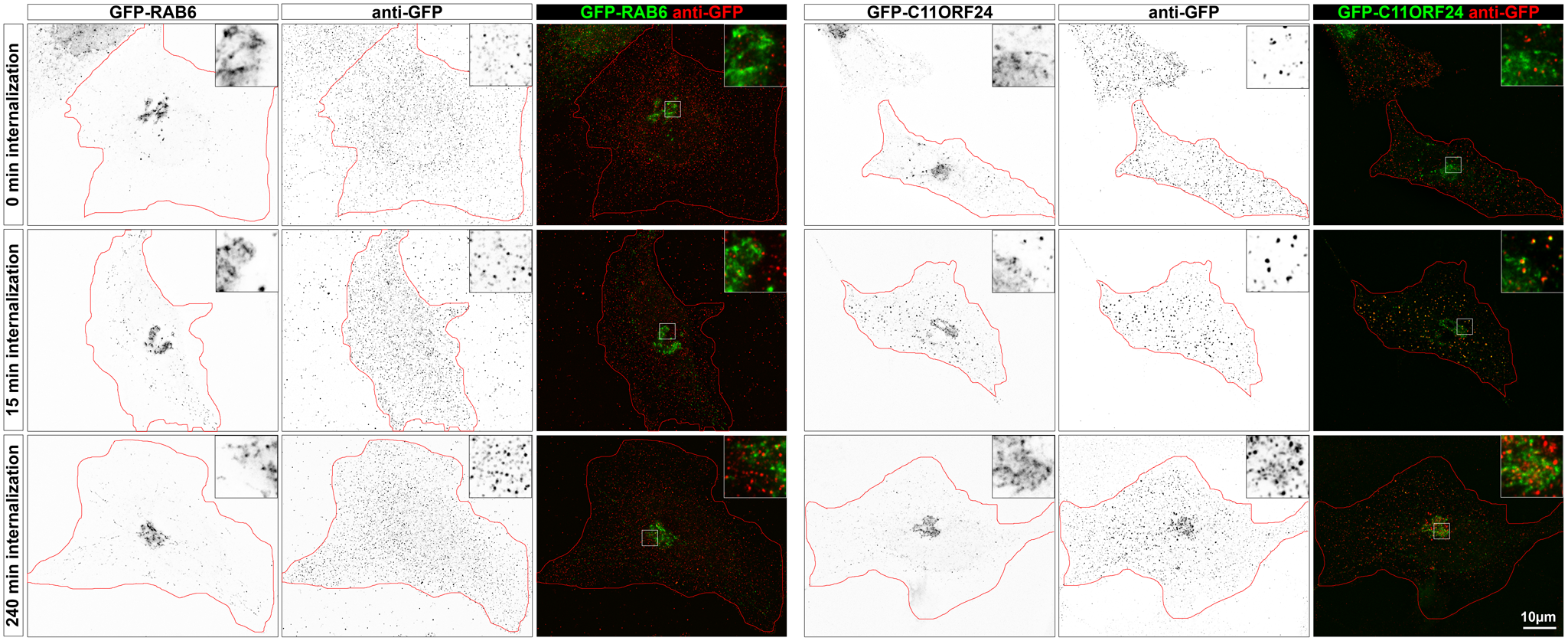

Supplement: Figure S3 — C11ORF24 is present at the plasma membrane and is internalized. HeLa cells were either transfected with GFP-RAB6 (left pannel) or GFP-C11ORF24 (right pannel) 18h prior to the incubation with the anti-GFP antibody and internalization was performed at 37°C for 0, 15 and 240 minutes. Cells were then fixed and stained with a secondary antibody and the localization of the anti-GFP antibody (red) was compared with the GFP signal (green). (TIF) [file pone.0082223.s003.tif]

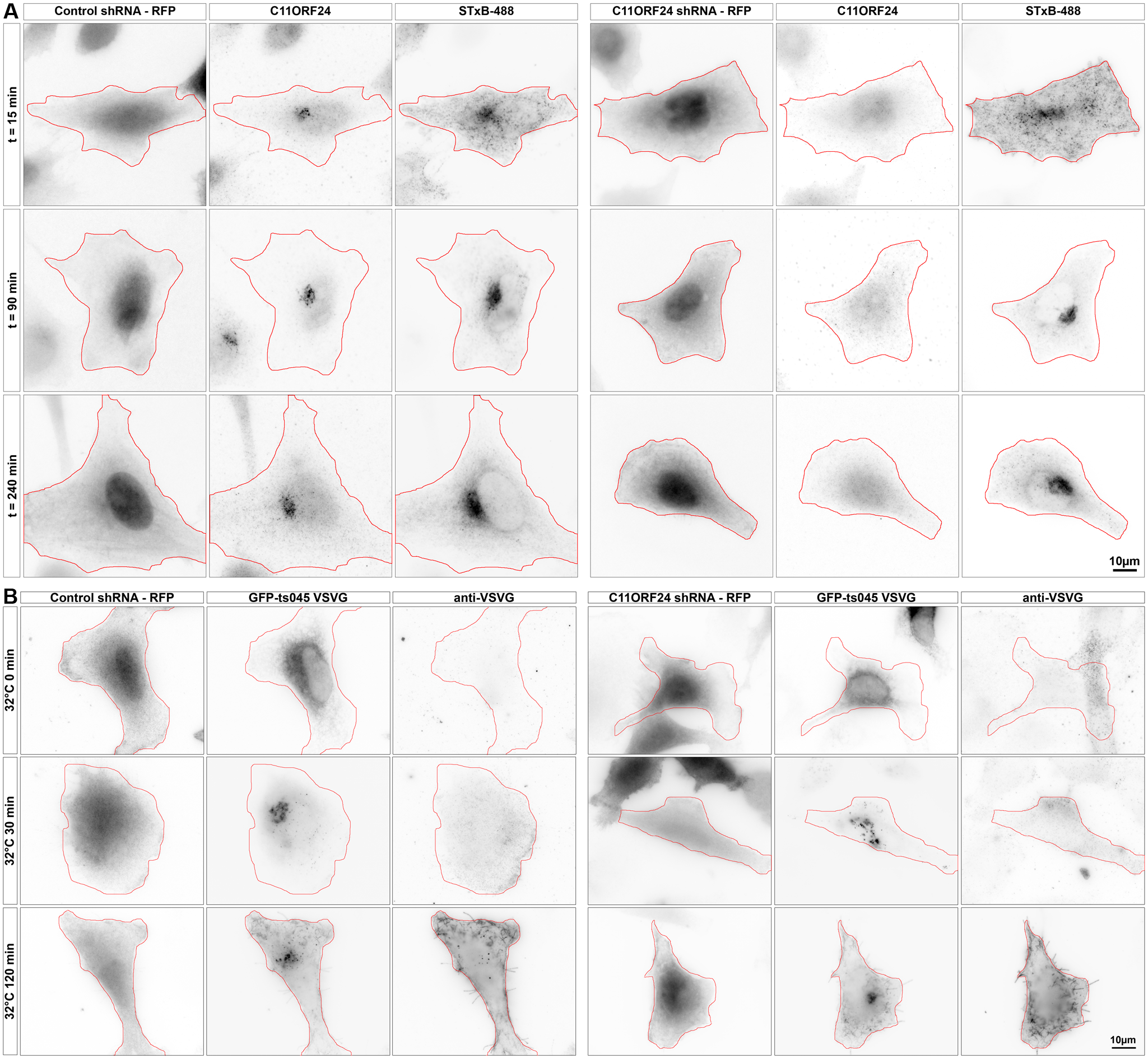

Supplement: Figure S4 — C11ORF24 is not necessary for the transport of classical cargos of the RAB6 pathway. (A) HeLa cells stably expressing either an inducible control shRNA or an shRNA against C11ORF24 were treated with doxycycline for 48 hours to induce shRNA and RFP expression. Cells were then incubated with STxB-488 for one hour at 4°C. Internalization was then performed at 37°C for the indicated times. Finally cells were fixed and stained with an anti-C11ORF24 antibody and the localization of STxB-488 over time was observed. (B) After a 48 hours knockdown cells were transfected with GFP-tsO45VSVG and incubated overnight at 40°C to retain the protein in the endoplamic reticulum and then switched to 32°C for 0, 30, 120 minutes to follow the secretion. Cells were then fixed and stained with and anti-VSVG antibody without permeabilization to detect specifically the arrival of the protein at the plasma membrane. (TIF) [file pone.0082223.s004.tif]

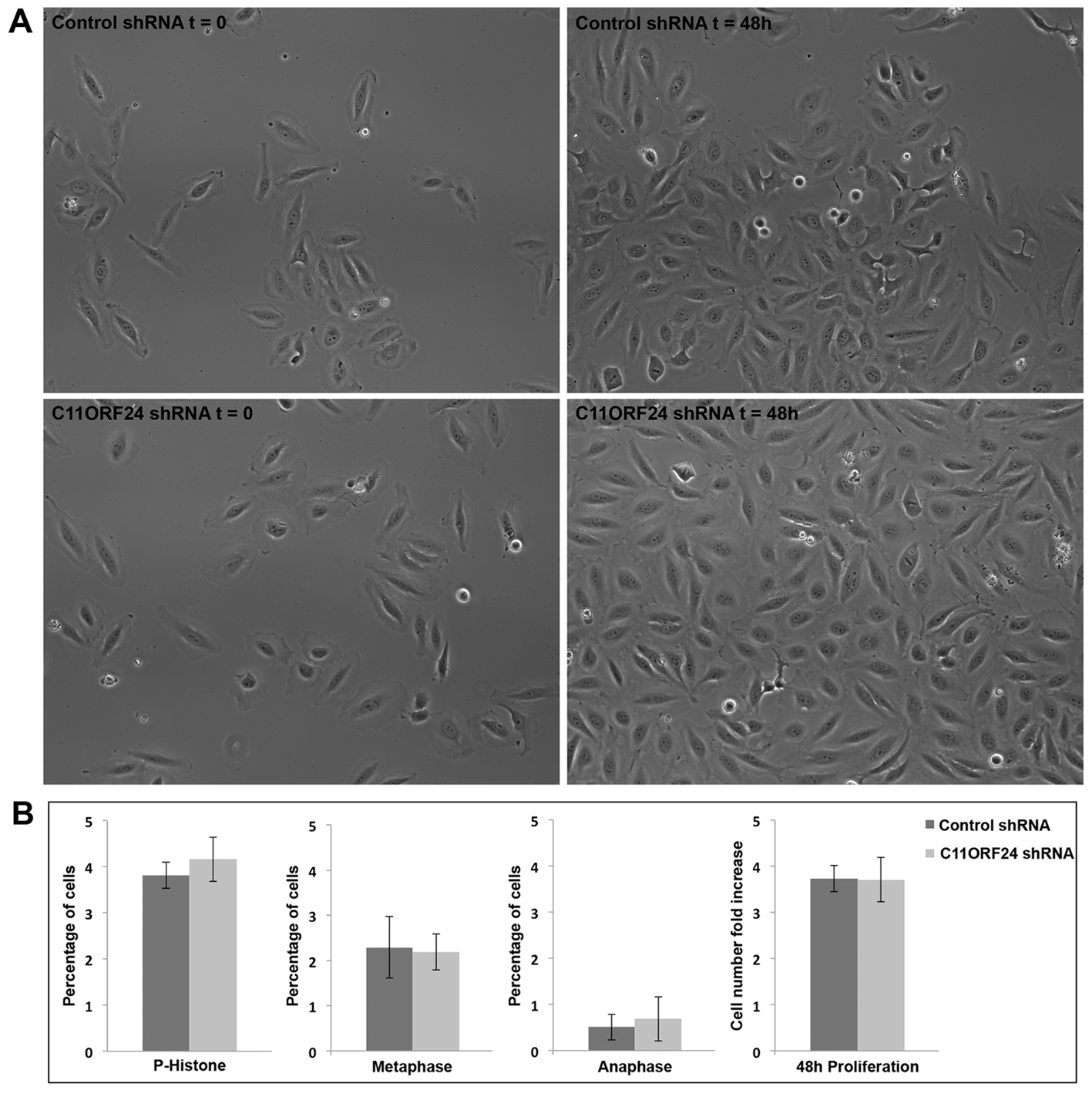

Supplement: Figure S5 — C11ORF24 is not necessary for cell cycle progression. HeLa cells stably expressing either an inducible control shRNA or an shRNA against C11ORF24 were treated with doxycycline for 48 hours to induce shRNA and RFP expression. A) Cells were then plated on microscopy chambers in the presence of doxycycline and observed every 10 minutes for 3 days by phase contrast. A snap shot of one representatives field is shown and the quantification is shown in B. B) Cells were fixed and stained with a phospho-histone antibody (P-Histone graph), DAPI to detect the DNA (Metaphase and Anaphase graphs), or counted during the movie shown in A (48h proliferation graph). (TIF) [file pone.0082223.s005.tif]
